# Matching algorithm for improving ride-sharing by incorporating route splits and social factors

**DOI:** 10.1371/journal.pone.0229674

**Published:** 2020-03-04

**Authors:** Omer Faruk Aydin, Ilgin Gokasar, Onur Kalan

**Affiliations:** 1 Department of Civil Engineering, Bogazici University, Istanbul, Turkey; 2 Department of Civil Engineering, Turkish-German University, Istanbul, Turkey; 3 Department of Civil and Urban Engineering, New York University, New York, NY, United States of America; Nanyang Technological University, SINGAPORE

## Abstract

Increasing traffic congestion and the advancements in technology have fostered the growth of alternative transportation modes such as dynamic ride-sharing. Smartphone technologies have enabled dynamic ride-sharing to thrive, as this type of transportation aims to establish ride matches between people with similar routes and schedules on short notice. Many automated matching methods are designed to improve system performance; such methods include minimizing process time, minimizing total system cost or maximizing total distance savings. However, the results may not provide the maximum benefits for the participants. This paper intends to develop an algorithm for optimizing matches when considering participants’ gender, age, employment status and social tendencies. The proposed matching algorithm also splits unmatched parts of drivers’ routes and creates new travel requests to find additional matches using these unmatched parts. Accordingly, this paper performs an extensive simulation study to assess the performance of the proposed algorithm. The simulation results indicate that route splits may increase the number of matches significantly when there is a shortage of drivers. Furthermore, the paper demonstrates the effects and potential benefits of utilizing a social compatibility score in the objective function.

## 1 Introduction and background

As traffic congestion worsens by the day, the rate of global warming accelerates as well accordingly. This situation has led to an increase in studies that aim to develop methods for reducing the use of private vehicles [1]. However, some studies have indicated a significant shift from public transportation toward private vehicles despite rising fuel prices [2]. While most vehicles can transport up to four passengers, the average passenger per vehicle ratio or private car occupancy rate in Europe was approximately 1.45 in 2015, specifically 1.42 in Germany, 1.38 in the Netherlands, and 1.58 in the UK [3]. Furthermore, traditional strategies in congestion management perspective, such as constructing new highways and maintaining current transportation infrastructure, requires expensive investment [4, 5]. Policy makers seeks alternative strategies (e.g. shared transportation) to reduce congestion. Consequently, ride-sharing may have great potential to ease traffic congestion.

Ride-sharing aims to bring together travelers with similar routes and schedules, and the idea is similar to the traditional dial-a-ride problem (DARP). The difference between ride-sharing and DARP is the type of driver supply; in a DARP, drivers are provided by a company within the DARP program, whereas drivers in a ride-sharing system are independent entities. In ride-sharing, drivers may have individual preferences and unique routes, and these factor can make the ride-sharing problem more complicated than a DARP. Rapid advances in technology in recent decades has promoted research into ride-sharing. The increasing use of smartphone devices and mobile applications has made ride-sharing more appealing than before [6–8]. The rise of ride-sharing can be traced back to the 1940s when it was done to conserve resources during World War II. At the time, the U.S. Office of Civilian Defense created a program called the “Car Sharing Club Exchange and Self-Dispatching System” to match riders and drivers via a bulletin board at their workplaces. The current era of ride-sharing includes the use of software packages, real-time services, financial incentives, and social networking platforms [9]. This has resulted in a dramatic increase in dynamic ride-sharing studies in recent decades [1, 10]. Dynamic ride-sharing requires an automated matching system; such a system can bring riders and drivers with similar travel patterns and schedules together on a very short notice. Dynamic ride-sharing systems are very complicated and require a great deal of attention from researchers, and therefore the success of a ride-sharing system depends on the successful implementation of ride-matching [10].

The success of a ride-sharing system also depends on having a sufficient number of participants and the feasible matches that would be accepted by participants. When participants repeatedly fail to find a match due to the lack of participants and trip announcements, they lose interest in the ride-sharing system and are very likely to stop their participation. It is shown that a constant rate of participation is required to achieve a sustainable ride-sharing system [7]. Therefore, especially in the start-up phase, it is crucial in order to attract enough participants to achieve a satisfactory matching rate. Some incentives can be suggested to attract more participants in the start-up phase; exploiting the potential capacities of drivers can also be considered.

There may be some other aspects that can help achieve success for a ride-sharing system. Many approaches in the literature require drivers to change their routes in order to increase system benefits, such as maximizing total distance savings or maximizing number of matched pairs. However, many drivers are not willing to change their routes because doing so may lead to a significant increase in their travel time. Likewise, long processing times cause long waiting times for users. Additionally, the characteristics and choices of users should be considered when attempting to implement a successful ride-sharing system in real-life. In the end, users consider only their own benefits, not system benefits [11].

This paper proposes a new ride-matching algorithm that aims to overcome the aforementioned challenges. Specifically, the paper intends to develop an algorithm that finds matches between riders and drivers on reasonably short notice by exploiting the capacities of drivers and by considering the characteristics and choices of the participants. In this regard, there are two main contributions of this paper to the literature. First, the paper discusses design and implementation of a ride-matching algorithm that finds more feasible matches by splitting a driver’s route. When a driver is matched with a rider for only part of the route, the unmatched part of the route is split, and a new travel request is created using the unmatched part so that the driver can be matched again with other riders. In this way, drivers can be matched more than once even if they have only one empty seat. An extensive simulation study is performed to assess the benefits of adding this feature to a ride-matching algorithm. The results suggest that this can increase the number of matches, especially when there is a small number of drivers. Secondly, the proposed algorithm includes the characteristics and choices of users, such as gender, age, employment, and their willingness to meet new people. Similar parameters have been presented in the literature as binary variables [12]. The proposed algorithm utilizes these parameters to find a common single parameter for scoring social compatibility between a rider and a driver. Using this approach, a rider can be matched with a driver even if some of the passengers’ choices are not completely satisfied, as long as the match is still acceptable.

The rest of the paper is structured as follows. Section “Related Studies” presents an overview of related studies, while Section 3 defines the problem and introduces the ride-sharing model. In Section 4, the solution approach for this ride-sharing model is outlined, and the application of the proposed algorithm is described. Section 5 discusses the details of the simulation study, and the results of the study are analyzed. Finally, Section 6 concludes the paper by summarizing the results of this study.

## 2 Related studies

In the recent decade, ride-sharing has receiving significant attention from both the transport and operation research community. In the literature, a number of studies on ride-sharing systems have identified the characteristics of ride-matching problems, and some have proposed solution methodologies [1, 13]. Traditional carpooling responds usually to recurring trips, such as home-based work trips [14], whereas ride-sharing is suitable for responding to non-recurring trips on a short notice, which is made possible due to the advance of communication technologies [7]. The non-recurring and short notice time period make finding matches for ride-sharing more difficult.

Dynamic ride-matching includes many parameters, and this renders the problem to be non-deterministic polynomial-time hard (NP-hard) [15–17]. Therefore, many solutions to the ride-matching problem that have been proposed in the literature use either heuristics or meta-heuristics [6, 15–23]. Although heuristic and meta-heuristic methods offer feasible processing times, they may not find the best possible matches.

To maximize system benefits, a previous study has proposed a novel approach to solve the ride-matching problem by modeling it using a traditional maximum-weight bipartite matching algorithm [7]. This algorithm is based on a single rider-single driver match. It is demonstrated that the weighted bipartite matching algorithm can be used for ride-matching, but this algorithm requires long processing times. The algorithm also omits matches of multiple riders with a single driver, and it ignores individual preferences in order to simplify the problem. Moreover, this algorithm assumes that a driver is willing to make a detour to pick up and drop off a rider, as long as the total distance saving is positive. This point suggests that the driver is willing to extend the trip time to increase system-wide benefits. It is clear that incentives such as cost allocation between riders and drivers may be helpful for matches to be accepted but results may not be satisfactory.

To increase the number of participants, one study introduced a rolling horizon approach in order to force the matching algorithm to postpone the finalization of the previously found matches until a deadline specified by the users [7]. This approach would not encourage people to be included in ride-sharing systems; the reason is that even if users specify a deadline for their travel request, they do not like to wait long [11]. Stiglic *et al*. [24] later extended the study by Agatz *et al*. [7] by adding meeting points to increase the number of matches. The algorithm here allows multiple riders-single driver matches if the riders are waiting at the same location. Another attempt of allowing a multiple riders-single driver match can be using a form of ride-sharing that is similar to carpooling, which considers sharing a ride from or to work [14]. One study proposed a non-exact evolutionary multi-objective ride-matching algorithm that allows for a multiple riders-single driver matches [17]. There are also studies in the literature that have modeled many-to-many ride matches. One study discussed allowing for transfers between different modes but did not offer a solution methodology [25]. Another study formulated a mixed integer problem to model many-to-many ride matches and offered heuristics to solve it [26]. A multi-hop ride-sharing system was also modeled as a binary optimization problem and the researchers introduced an algorithm to solve this problem [6]. Another study even considered employing dedicated drivers in order to achieve a satisfactory number of drivers in the start-up phase [22].

Many studies in the literature have employed a number of objectives to solve ride-matching problems. These include maximizing total distance savings [7, 27], minimizing travel distances [28], maximizing fuel savings [29], maximizing number of matches [11, 24, 27], minimizing total system travel costs [30, 31], and minimizing travel times [32]. These objectives are generally proportional to each other, and they mostly focus on system-wide benefits.

The characteristics and the choices of participants are very important for the participants as they decide whether the match found by an algorithm is reasonable by means of social compatibility. Thus, participants’ choices are incorporated into optimization algorithms. In previous studies, the trip preferences of both drivers and riders were incorporated as constraints, and such preferences include age, gender, smoking preference, and even pet restrictions; however, these are not used in the objective functions [12, 18, 33]. In the algorithm created by Ghoseiri *et al*. [12], each rider’s choices are compared to the characteristics of the potential driver and the characteristics of other riders who travel with that driver. The algorithm uses binary decision variables to accept or reject a match. Thereafter, one study introduced the stable match concept, and it examined the trade-off between matches that consider the individual benefits of users and matches that consider the benefits of the system as a whole [34]. This study examined the trade-off between matches that consider the individual benefits of users and matches that consider the benefits of the system as a whole.

In contrast with existing studies, this paper considers social characteristics and choices of participants and utilizes them in the objective function; thus, more people may be willing to participate in a ride-sharing system in real life. In the literature, there is one study that utilized social parameters in their algorithm; however, they set these parameters as decision variables that may cause a significant decrease in the number of matches. In this paper, a new parameter for scoring social compatibility is introduced to address this challenge. Using a new parameter, the social compatibility of participants are considered for the matching process, and consequently there would be no loss of matches that may occur when social parameters are given as constraints. To achieve critical mass in ride-sharing, various solution approaches, such as rolling horizon, many-to-many matches, employing dedicated drivers, are proposed in the literature; however, this problem has not yet been fully resolved. To overcome this challenge, in this paper, capacities of drivers are exploited by splitting their routes after they are matched with a rider so that the unmatched part of the route can be utilized for other riders. Thus, the possibility of finding satisfactory number of drivers to maintain a sustainable ride-sharing system can be increased.

## 3 Problem definition

The ride-sharing system contains a set of participants *P*. These participants are divided into two groups: a set of drivers *D* and a set of riders *R*. Each rider and driver make a trip announcement, which is defined as their travel requests. A set of trip announcements *S* is defined such that *R* ⊂ *S* and *D* ⊂ *S*. Each trip announcement *s* ⊂ *S* is associated with origin and destination locations *o*_*s*_ and *d*_*s*_.

A set of meeting locations *M* is defined. The feasible routes *U*_*ij*_ from location *i* to *j* are known and constant, where *i*, *j* ∈ *M*. Travel distance *d*_*ij*_, travel time *t*_*ij*_, and meeting locations on the route *k*_1,2,…,*n*_ are defined for each route *u*_*ij*_ ∈ *Uij*. A route can be chosen by a driver or the shortest route can be calculated and assigned for the driver. Routes are determined when drivers send a travel request, and it is assumed that drivers will not change the route.

For each driver, the system defines a travel announcement time *T*^*A*^, (i.e., the time a participant send a travel request to the system) and the arrival time *T*_*k*_ to a location *k*. The latest departure time from origin $T_{o}^{LD}$ and the latest arrival time to destination location $T_{d}^{LA}$ are defined for each rider.

In this algorithm, each rider *r* ∈ *R* specifies the rider count *c*_*r*_, which refers to the number of riders willing to travel together as a rider group. For example, a single rider’s rider count value is 1, whereas two friends who are willing to travel together in the same vehicle have a rider count value of 2. Each driver *d* ∈ *D* specifies his or her capacity, as in the number of empty seats *c*_*d*_. A novel aspect of this algorithm is the objective function, which maximizes the participants’ benefits by considering their characteristics and choices. Shaheen *et al*. [35] suggested that gender, age, and employment status are key drivers of ride-sharing. With this in mind, the proposed algorithm uses four parameters (gender *g*_*s*_; age *a*_*s*_; employment status *w*_*s*_; socialness or willingness to meet new people, *δ*_*s*_) as well as their respective weights (gender weight *γ*_*g*_; age weight *γ*_*a*_; employment status weight *γ*_*w*_; and socialness weight *γ*_*δ*_) to define the benefits of the participants. Using these social parameters and their weights, a joint socialness score (JSS) is defined to score the similarity between a driver *d* and a rider *r* as follows:

$$\begin{array}{c} \gamma^{rd}=x_{g}^{rd}\gamma_{g}^{r}\gamma_{g}^{d}+x_{a}^{rd}\gamma_{a}^{r}\gamma_{a}^{d}+x_{w}^{rd}\gamma_{w}^{r}\gamma_{w}^{d}+x_{\delta }^{rd}\gamma_{\delta }^{r}\gamma_{\delta }^{d}. \end{array}$$ (1)

In Eq 1, the weights of the social factors of the rider *r* and a driver *d* who is feasible for the rider are multiplied to calculate the JSS *γ*_*rd*_. The variable *x* is defined such that *x* ∈ {−1, 1}. Its value is +1 if the social characteristics are the same and −1 if they are different. A sample calculation of the JSS is presented in Table 1, which presents the characteristics of driver *d*1 and rider *r*1. It is assumed that all the participants want to be matched with a participant with similar characteristics. The social factor weights are obtained from the participants, who are asked to rate the weights of each social factor from 0 to 5. A rating of 0 indicates that it is not important to be matched with a user with the same social characteristic, while a rating of 5 indicates that being matched with a similar user is very important.

**Table 1:** An illustrative example of the computation of the JSS.

	Driver *d*1		Rider *r*1			
	Characteristics	Factor	Characteristics	Factor	*x*_*rd*_	Scores
Gender	male	1	female	5	-1	-5
Age	18-25	3	25-40	4	-1	-12
Employment	TAU	4	TAU	4	1	16
Socialness	Yes	5	Yes	3	1	15
Total score						14

In the example given in Table 1, driver *d*1 is a male driver aged between 18 to 25 years who works at the Turkish-German University (TAU). Driver *d*1 stated that the weights of a rider’s gender, age range, and working place are scored 1, 3 and 4 out of 5, respectively. Driver *d*1 also stated that he is willing to meet new people with a weight factor of 5. By contrast, rider *r*1 is a female aged between 25 to 40 years who also works at TAU. The weight factor for her willingness to meet new people is 3. As mentioned previously, the variable *x*_*rd*_ is assigned a value of +1 if characteristics are the same and −1 otherwise. In this situation, the value of *x*_*rd*_ is −1 for gender and age because the driver and the rider’s gender and age range are different. The value of *x*_*rd*_ is +1 for both employment and socialness because they are working at the same location and are willing to meet with new people. The score for gender equals to 1*x*5*x*(−1) = (−5). When the scores of the other social characteristics are calculated in this way, the JSS can be calculated by simply adding all the scores.

The aggregation of social parameters into one parameter would imply some of the social factors would be sacrificed for the benefit of others, but such concerns can be eliminated easily by adding some constraints for each social characteristic. The reason of utilizing one parameter, namely JSS, is to maintain the maximum number of participants in a ride-sharing system. When constraints for each parameter are utilized, many matches would be eliminated. The assumption is that if there is no better match, a participant would accept a match with another participant even if some characteristics do not match. Nevertheless, participants would like to be matched with their most compatible participants in the system.

### 3.1 Feasible match

A match between a rider and a driver can be considered feasible if their routes and schedules are similar. These similarities are defined as spatial and temporal constraints. Since it is assumed that a driver *d* will pick up a rider *r* on his or her route, the origin and destination of rider *r* must be on the driver’s route, and they must travel in the same direction. Thus, a match between driver *d* and rider *r* is spatially feasible if the following equations are satisfied:

$$\begin{array}{c} o_{r},d_{r}\subset u_{d}, \end{array}$$ (2)

$$\begin{array}{c} T_{o_{r}}\left(d\right)\leq T_{o_{d}}\left(d\right). \end{array}$$ (3)

Eq 2 ensures that the rider’s origin *o*_*r*_ and destination *d*_*r*_ located on the driver’s route *u*_*d*_, and Eq 3 states that the driver visits the rider’s origin before the rider’s destination; thus, it is ensured that they are traveling in the same direction.

To check the time feasibility, it is assumed that riders will wait past their latest departure time as long as they know a driver is coming for them. At the same time, a constraint for waiting time *w* can be set as well. When driver *d* sends a travel request with a departure time and a route, the time feasibility between driver *d* and rider *r* can be checked such that

$$\begin{array}{c} T^{A}\left(d\right)\leq T_{o}^{LD}\left(r\right), \end{array}$$ (4)

$$\begin{array}{c} T_{k}\left(d\right)\leq T_{d}^{LA}\left(r\right)-t_{o_{r}d_{r}}, \end{array}$$ (5)

$$\begin{array}{c} T_{k}\left(d\right)\leq T^{A}\left(r\right)+w. \end{array}$$ (6)

Eq 4 states that the travel announcement of driver *d* *T*^*A*^(*d*) is made before the latest departure time of rider *r* from the origin $T_{o}^{LD}(r)$. Eq 5 ensures that if driver *d* picks up rider *r*, they will reach destination of the rider before the rider’s latest arrival time (i.e., travel time from rider’s origin to destination $t_{o_{r}d_{r}}$ added to the time of the driver’s arrival to meeting point *T*_*k*_(*d*) should not exceed the rider’s latest arrival time $T_{d}^{LA}(r)$). Eq 6 states that rider *r* will wait for a driver up to the maximum waiting period *w* after the travel announcement time *T*^*A*^(*r*).

A feasible match must also satisfy the capacity constraint.

$$\begin{array}{c} \begin{array}{cc} & c_{d}\geq c_{r}. \end{array} \end{array}$$ (7)

Eq 7 implies that the number of empty seats available in a driver’s vehicle *c*_*d*_ should be greater than or equal to the rider count *c*_*r*_ (i.e., number of riders willing to travel as a group by sending a single travel request).

### 3.2 Matching problem

Ride-matching algorithms in the literature have utilized a variety of objective functions and constraints. One of the most popular objective function is maximizing travel distance savings. Travel distance saving is the difference between the distance traveled when participants share a ride and when they travel by themselves. When a driver picks up a rider at the rider’s origin and drops off the rider at the rider’s destination, the travel distance saving *σ*_*dr*_ is calculated such that

$$\begin{array}{c} \sigma_{dr}=d_{o_{d}d_{d}}-\left(d_{o_{d}o_{r}}+d_{o_{r}d_{r}}+d_{d_{r}d_{d}}\right)+\sum_{r\in R}\left(d_{o_{r}d_{r}}\right). \end{array}$$ (8)

When drivers are not willing to change their routes (i.e., only accepts to be matched with riders located on their routes), Eq 8 equals to the following:

$$\begin{array}{c} \sigma_{dr}=\sum_{r\in R}\left(d_{o_{r}d_{r}}\right). \end{array}$$ (9)

Travel distance saving is directly proportional to many other objective functions, such as travel time saving and fuel saving. In this paper, maximizing a new parameter, namely JSS in Eq 1, is introduced as an objective function.

**Fig 1:**
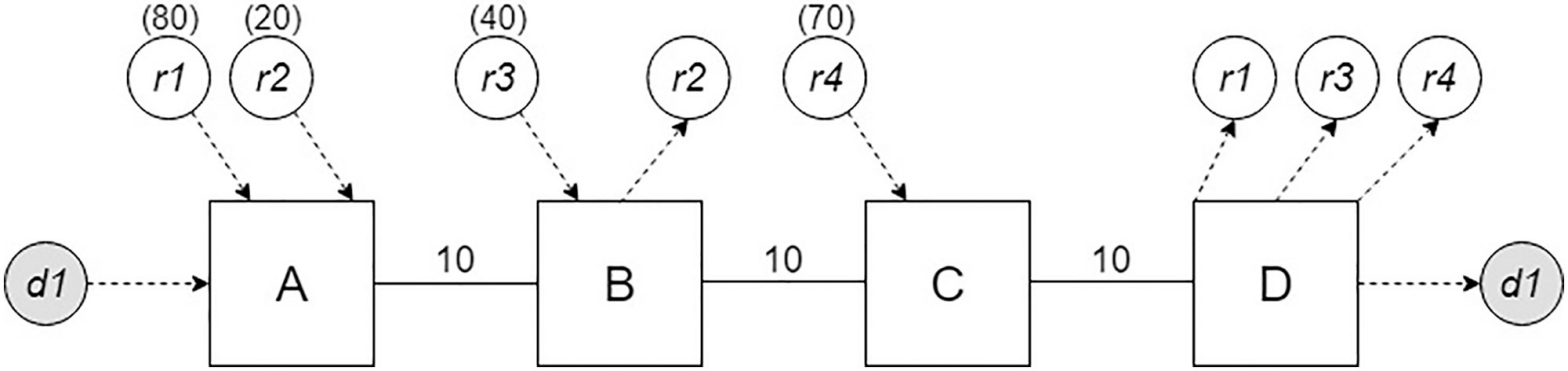
Ride-sharing schema for a driver and four riders.

**Fig 2:**
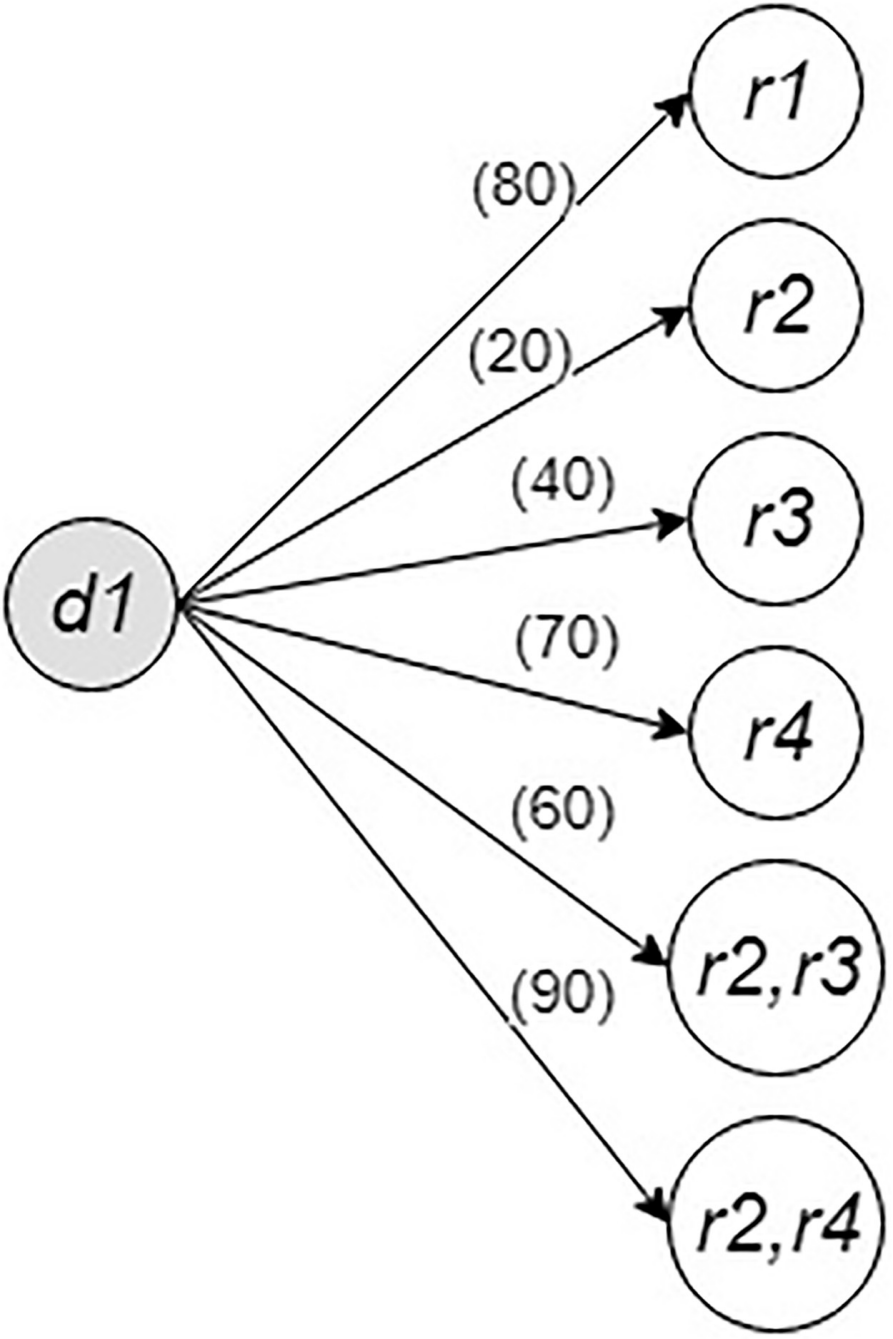
Bipartite graph for a driver and four riders.

The example given in Fig 1 shows driver *d*1 and four riders, namely *r*1, *r*2, *r*3, and *r*4. The letters A, B, C, and D represent locations. In this example, driver *d*1 has origin and destination locations A and D, respectively. The travel requests from the riders are as follows: rider *r*1 from A to D, rider *r*2 from A to B, rider *r*3 from B to D, and rider *r*4 from C to D. The numbers in the parentheses show the JSS between a rider and driver *d*1, and the numbers on the links connecting locations represent travel distances. The ride-matching problem is to decide which rider or riders should be matched with driver *d*1. The result can be affected by an objective function and/or constraints. Furthermore, the solution approach may affect the results as well. All four riders in this example can be matched with driver *d*1 separately. In addition, two sets of riders, namely riders *r*2 with *r*3 and riders *r*2 with *r*4, can create feasible pairs with driver *d*1 if the driver *d*1 has a single available seat. The bipartite graph for this example is shown in Fig 2. In Fig 2, the nodes represent participants and links represent edges created for feasible matches. The numbers in parentheses on the links show the JSSs of the matches represented by the links. The outcomes of these possible matches are given in Table 2.

**Table 2:** Outcomes of the possible matches in a ride-sharing schema.

Scenario #	Match	No. of matches	No. of extra stops	JSS	Distance savings	Optimal Solution		Heuristic Solution	
						Obj.: Max JSS	Obj.: Max distance savings	Obj.: Max JSS	Obj.: Max distance savings
1	d1 → r1	1	0	80	30		x	x	x
2	d1 → r2	1	1	20	10				
3	d1 → r3	1	1	40	20				
4	d1 → r4	1	1	70	10				
5	d1 → r2, r3	2	1	60	30		x		
6	d1 → r2, r4	2	2	90	20	x			

In the example, an optimization model with an objective function of maximizing JSS finds Scenario 6 to be an optimal solution, while the objective function of maximizing distance savings seems to fit either Scenario 1 or 5. When a heuristic approach is proposed, Scenario 1 would be chosen for both the objective functions of maximizing JSS and distance savings. Moreover, adding some constraints such as a lower bound for JSS or number of stops made by drivers would change the optimal match.

A node for each driver *d* ∈ *D* and each rider *r* ∈ *R* is defined. For each feasible match, an edge is created between the driver and the rider. The ride-sharing system presented in this paper offers the possibility that a driver can be matched with more than one rider even if only single rider-single driver matches are allowed. This can be achieved by introducing extra nodes that represent a set of riders *J*. To determine which riders can create a joint group to be matched with the same driver, a subproblem can be created such that

$$\begin{array}{c} t_{o_{r1}d_{r1}}+t_{d_{r1}o_{r2}}+t_{o_{r2}d_{r2}}\leq t_{o_{r1}d_{r2}}. \end{array}$$ (10)

$$\begin{array}{c} o_{r1},d_{r1},o_{r2},d_{r2}\subset u_{d}. \end{array}$$ (11)

Assuming constant travel times, Eq 10 ensures that the origin of rider *r*2 is visited by driver *d* after the destination of rider *r*1 is visited so that they can be picked up by the same driver. Eq 11 ensures that the origins *o*_*r*1_, *o*_*r*2_ and destinations *d*_*r*1_, *d*_*r*2_ of both riders *r*1 and *r*2 are located on driver’s route *u*_*d*_ so that the match can be feasible. Both Eqs 10 and 11 hold for a set of two riders, and the equations can be expanded for more than two riders if needed.

Each edge *e* is assigned with a JSS or distance saving depending on the objective function. *E* represents a set of all edges, and *y*_*e*_ is a binary decision variable for *e* ∈ *E*; *y*_*e*_ is assigned a value of 1 if the edge is an optimal match and 0 otherwise. *E*_*d*_ and *E*_*r*_ represent the set of edges associated with driver *d* and rider *r* respectively. In this way, the ride-match between a driver with one empty seat and multiple riders with the objective of maximizing JSS can then be formulated as the following integer program:

$$\begin{array}{c} max\;z_{1}=\sum_{e\in E}\left(\gamma_{e}^{rd}+100\right)y_{e} \end{array}$$ (12)

subject to

$$\begin{array}{c} \sum_{e\in E_{d}}y_{e}\leq 1\;\forall d\in D, \end{array}$$ (13)

$$\begin{array}{c} \sum_{e\in E_{r}}y_{e}\leq 1\;\forall r\in R, \end{array}$$ (14)

$$\begin{array}{c} y_{e}\in \left\{0,1\right\}\;\forall e\in E. \end{array}$$ (15)

Eq 12 represents the objective of maximizing the sum of JSSs $\gamma_{e}^{rd}$. A constant of 100 is added to the JSS so that the matches with negative JSSs will not be eliminated in order to maximize the objective function. Eqs 13 and 14 ensure that a driver and a rider or a rider set are included in only one optimal solution. Eq 15 shows that *y*_*e*_ is a binary variable that is assigned for each edge. The objective function can be easily changed to maximizing distance savings by replacing Eq 12 with the following equation:

$$\begin{array}{c} max\;z_{2}=\sum_{e\in E}\sigma_{e}y_{e} \end{array}$$ (16)

The problem can be extended by adding more constraints, such as setting a lower bound for JSS or limiting number of stops made by a driver to pick up or drop off a rider. Furthermore, constraints for each social parameter can be utilized at will in order to maintain a certain level of quality for the matches.

## 4 Solution approach

One of the most significant barriers in ride-matching problems is dealing with a large number of participants within a feasible time period [1]. The integer programming (IP) formulation described in Section 3 is computationally prohibitive in solving for large-scale instances. When multiple drivers and the presence of joint riders are considered, it is necessary to use a heuristic that is capable of solving large-scale instances in feasible times. This section discusses the approach that is adopted to solve the defined ride-matching problem.

### 4.1 Route feasibility

Most traditional weighted bipartite matching algorithms in the literature assumed that drivers are willing to change their routes to pick up and drop off riders. Accordingly, the route feasibility condition holds if the travel distance savings is positive. In the proposed algorithm, drivers’ routes are assumed to be specified by drivers when they send ride-share requests. Therefore, to satisfy the route feasibility constraint, a rider’s origin and destination locations should be on the driver’s route. In addition, it must be ensured that the directions of the rider and driver are the same, meaning that alignment of their routes must be checked.

To check the similarities between the routes of the drivers and riders, this paper uses the Needleman-Wunsch algorithm, which is one of the first examples of dynamic programming. The Needleman-Wunsch algorithm scores the alignment of two groups of letters [36]. This means that the Needleman-Wunsch algorithm can be used not only to check the origin and destination locations, but also the midpoints specified by participants. Furthermore, the algorithm also checks the order of the letters. This algorithm results in a high quality alignment. To date, it is widely used in the bioinformatics field to identify similarities between a sample amino acid chain with amino acid chains recorded in a database [37].

Using the algorithm, a matrix (M) is created, and the scores of *matching*, *mismatching*, and *gap* are specified at will. These scores are assigned to the cells such that (a) if the letters in the corresponding column and row are the same, a matching score is assigned; (b) if they are different, a mismatching score is assigned; and (c) if one of the letters is missing, gap score is assigned. The algorithm has various solving methods, but all of them give the same result. The steps involved in solving the problem are as follows [38]:

1. A matrix S is defined, where *i* and *j* denote the row and column numbers. Let *m* and *n* denote the lengths of the first and second letter arrays, then 0 ≤ *i* ≤ *m* and 0 ≤ *j* ≤ *n*.
2. The values of S are set to 1 if there is a match and to 0 if there is no match (assuming the matching score is 1 and the mismatching score is 0). If there is a gap in the letter groups, a gap score is assigned. When the gap score is 0, *S*[*i*, 0] = 0 for *i* = 0, 1, …, *m* and *S*[0, *j*] = 0 for *j* = 0, 1, …, *n*.
3. Compute the scores starting from the top-left cell using following equation:
  $$\begin{array}{c} \begin{array}{cc} & M[i,j]=S[i,j]+max(M[i-1:x],M[j-1:y]). \end{array} \end{array}$$ (17)
4. Start the traceback process from the bottom-right cell and continue by selecting the cell with the lowest value from the adjacent columns and rows.

As an example, let driver *d* defines a route “ABCDE” and rider *r* defines an origin C and destination E. The sequence alignment for these letter arrays will result in a score of 2, and an alignment is found such that

$$\begin{array}{c} \begin{array}{ccc} AB & CD & E\\ -- & C- & E. \end{array} \end{array}$$

**Fig 3:**
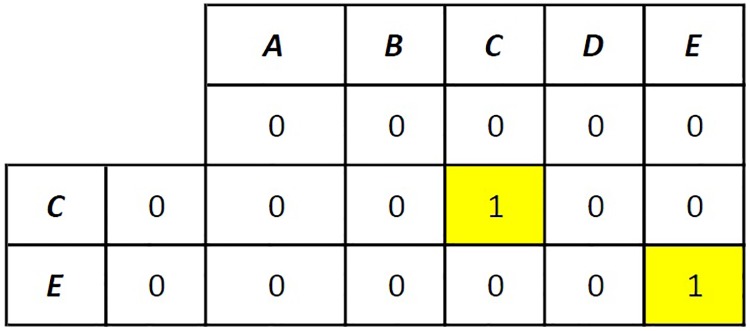
Needleman-Wunsch algorithm after the generation of the S matrix.

**Fig 4:**
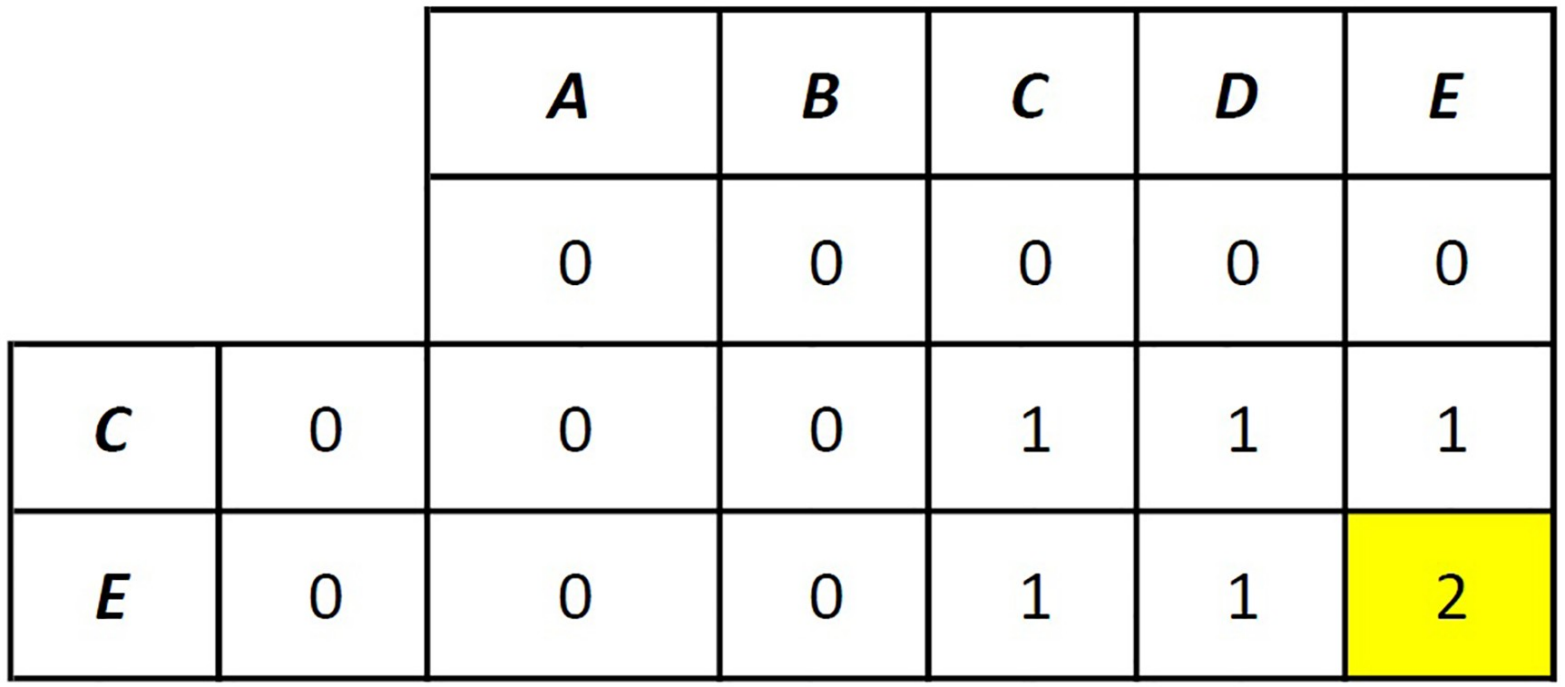
Needleman-Wunsch algorithm after the generation of the M matrix.

The algorithm proposed in this paper, only needs the matching letters and their order, not the gaps between the letters. Therefore, the Needleman-Wunsch algorithm is modified by eliminating the traceback process. In this algorithm, the calculation of the matrix begins at the top-left cell and finishes at the bottom-right cell. The S and M matrices are calculated as depicted in Figs 3 and 4. In Fig 3, the S matrix is created as follows: If the letters are the same, then a matching score of 1 is written; otherwise, a mismatching score of zero is written. In Fig 4, the M matrix is created using Eq 17. Since S[1, 1] = 0, M[0, 1] = 0 and M[1, 0] = 0, M[1, 1] is calculated as 0. The bottom-right cell M[2, 5] is calculated as 2 because S[1, 5] = 1, M[1, 5] = 1 and M[2, 4] = 1, thus max(M[1, 5], M[2, 4]) = 1 and M[2, 5] = S[2, 5] + 1 = 2.

When using the Needleman-Wunsch algorithm for route checking, if the letters representing the origin and the destination of the rider are present along the route of the driver in order, the score (i.e., the value of the cell *mxn*) equals 2. It should be noted that when “ABCDE” is compared with “EC,” which is in the opposite direction, the score equals to 1. Thus, it is concluded that when the Needleman-Wunsch algorithm is used for comparing the letter arrays that represent the route of driver *d* and the origin and destination of rider *r* and when the matching score is 1, driver *d* and rider *r* are said to be spatially feasible, if the score is 2. The proposed algorithm for checking route feasibility is given in Algorithm 1.

**Algorithm 1** The Needleman-Wunsch Algorithm to check route feasibility.

*m* = number of letters in rider’s route

*n* = number of letters in driver’s route

*matchscore* = 1

**for** i in range (0, m+1) **do**

 *score*[*i*][0] = 0

**end for**

**for** j in range (0, n+1) **do**

 *score*[0][*j*] = 0

**end for**

**for** i in range(1, m+1) **do**

 **for** j in range(1, n+1) **do**

  *match* = *score*[*i* − 1][*j* − 1] + *matchscore*(*seq*1[*i* − 1], *seq*2[*j* − 1])

  *score*[*i*][*j*] = *match*

 **end for**

**end for**

**return** *score*[*m*][*n*]

### 4.2 Splitting drivers’ routes and recording extra stops

When it is assumed that drivers will detour to pick up and drop off riders, a driver can be matched with a single rider or with multiple riders who have the same origin and destination because the route is determined according to riders’ routes. In the algorithm proposed in this paper, a driver will only engage with riders located along the driver’s route. When the driver is matched with a rider, his or her capacity is decreased for the whole route, even if the driver is matched with a rider who is sharing only a small part of the route. To overcome this challenge, when a driver is matched, a new travel request is created using an unmatched part of the route. Accordingly, the driver needs to make extra stops to pick up and drop off riders, if they have different origins or destinations. If drivers have larger vehicle capacities and longer routes, they would need to make even more extra stops. To prevent this inconvenience for drivers, extra stops should be recorded so that they can be limited. The proposed approach for splitting drivers’ routes and recording extra stops is given in Algorithm 2.

**Algorithm 2** Creating new request by splitting drivers’ routes and recording extra stops.

**for** each driver **do**

 add ID, origin and destination of driver, i.e. (*ID*, [*o*_*d*_, *d*_*d*_]) to the *stops* list

**end for**

(*r*, *d*) = the current best match having route, time and capacity feasibility

**if** *o*_*r*_, *d*_*r*_ **not in** *stops* **and** (*number of stops visited by d*) + 2 > *limitforstops*+2

**then**

 select the next best match

**else**

 **if** *o*_*r*_ **not in** *stops* **and** (*number of stops visited by d*) + 1 > *limitforstops*+2

 **then**

  select the next best match

 **end if**

 **if** *d*_*r*_ **not in** *stops* **and** (*number of stops visited by d*) + 1 > *limitforstops*+2

 **then**

  select the next best match

 **end if**

**end if**

**if** *o*_*r*_! = *o*_*d*_ **then**

 **if** *o*_*r*_ **not in** *stops visited by d* **then**

  add *o*_*r*_ to the stops visited by *d*

 **end if**

 create a new request with driver *d*

 update capacity of the new request with rider count of *r*, *c*_*r*_

 update destination of the new request with *o*_*r*_

 send the new request to the database

**end if**

**if** *d*_*r*_! = *d*_*d*_ **then**

 **if** *d*_*r*_ **not in** *stops visited by d* **then**

  add *d*_*r*_ to the stops visited by *d*

 **end if**

 create a new request with driver *d*

 update capacity of the new request with rider count of *r*, *c*_*r*_

 update origin of the new request with *d*_*r*_

 send the new request to the database

**end if**

In the example depicted in Fig 1, when driver *d*1 is matched with rider *r*2, driver *d*1 picks up rider *r*2 at point A, which is the origin of both driver *d*1 and rider *r*2; the driver then drops off rider *r*2 at destination B, which is different from driver’s destination. A new travel request from *d*1 can be created for the route from B to D. Algorithm 2 creates this new travel request and records location B as an extra stop.

### 4.3 Matching process

**Fig 5:**
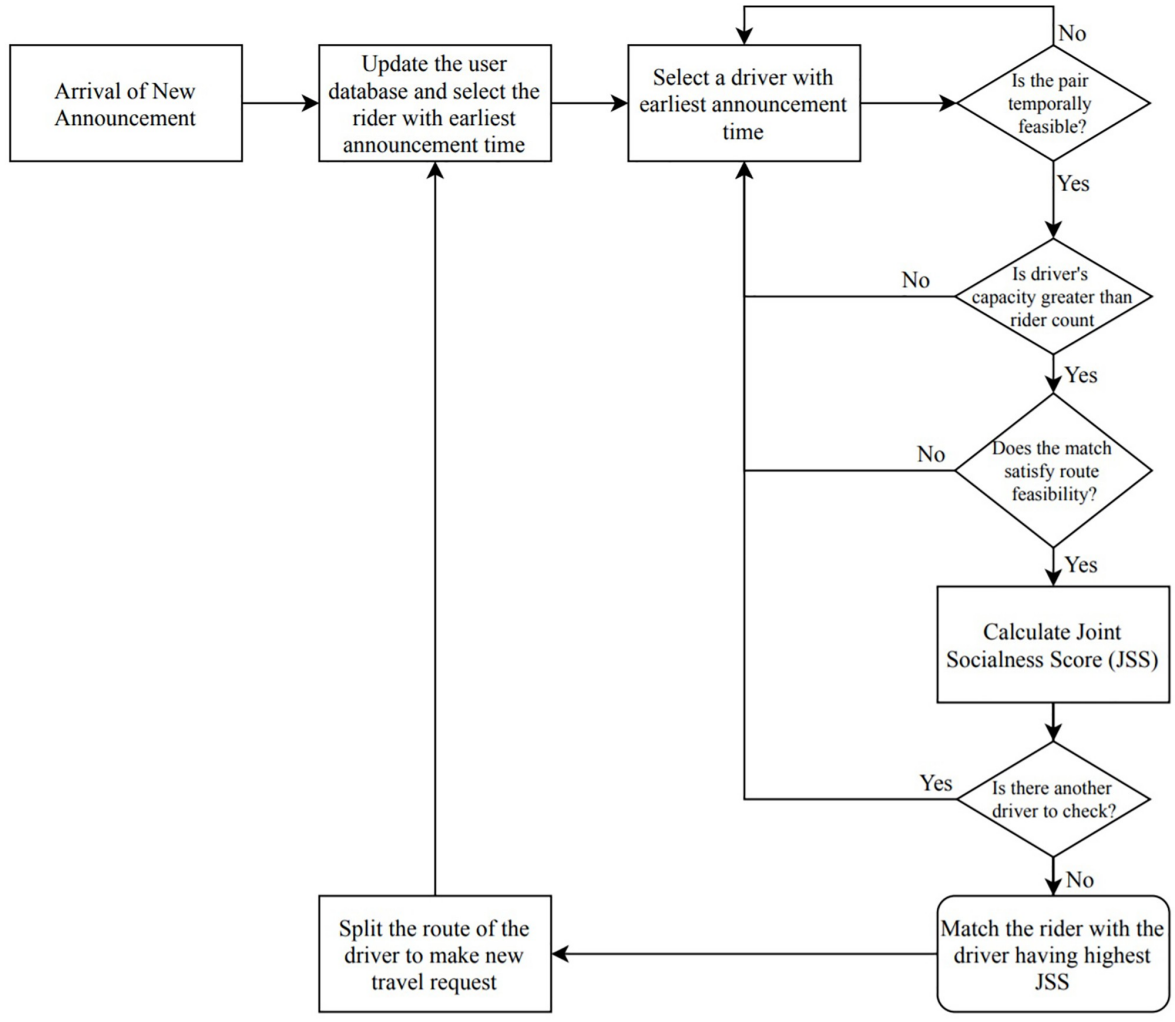
Flowchart of the matching process.

In this section, the matching process is outlined. The matching process is carefully constructed to ease the computational burden it imposes on the systems used. The first-come-first-serve method is applied: when a rider enters the system, the feasibility of each available driver is first checked. After this, the JSSs for all feasible drivers are calculated. The rider is matched with the driver whose corresponding JSS is the highest. The steps of the proposed algorithm are illustrated as a flowchart in Fig 5; the matching algorithm is described in Algorithm 3. These steps are as follows:

1. If there is a new announcement, update the database.
2. Select the unmatched rider whose announcement time is the earliest.
3. Select the temporally feasible driver with the earliest announcement time.
4. Check the capacity, time and route feasibility of a match between the rider and driver.
5. If the driver is feasible for the rider, calculate the JSS between them and add this pair to the feasible matches list.
6. If there is an unchecked driver, go to Step 3 and repeat the process.
7. Select the driver with the best JSS from the feasible matches list and match the driver with the rider.
8. Eliminate the rider from the system. Subtract the rider count from the capacity of the matched driver and update the capacity of the driver.
9. If driver’s origin and/or destination are different from that of the rider’s, split the driver’s route to create a new travel request for the unmatched parts of the route.
10. Update the database and repeat the process, starting from Step 1.

**Algorithm 3** The matching process.

**for** each rider **do**

 **for** each driver **do**

  **if** time is feasible **then**

   **if** capacity >= rider count **then**

    **if** route is feasible **then**

     calculate JSS

     add match to the *feasiblematches* list

    **end if**

   **end if**

  **end if**

 **end for**

 select the match with the highest JSS from *feasiblematches* list

 match the rider and eliminate the rider from the system

 update driver capacity

 create new travel request with the driver and record the stops

 update the database

**end for**

In Algorithm 3, a rider is selected and for this rider each driver is analyzed one-by-one. Firstly, temporal compatibility of this couple is checked. If they are temporally compatible, capacity constraints and route feasibility are checked using Eq 7 and Algorithm 1, respectively. When these constraints are satisfied, the JSS of this match is calculated using Eq 1 and this match is added to the list of feasible matches. When all drivers are examined for the first rider, the rider is matched with the driver having maximum JSS. Later, the rider is eliminated from the system and the route of the matched driver is split and capacity is updated using Algorithm 2. The next rider is selected and processes are repeated until all riders are checked.

## 5 Computational study

In this section, a comprehensive simulation study is conducted to assess the potential benefits of creating additional travel requests by splitting drivers’ routes. The paper also examines the effects of using JSS in the objective function.

### 5.1 Data generation and experiments

**Fig 6:**
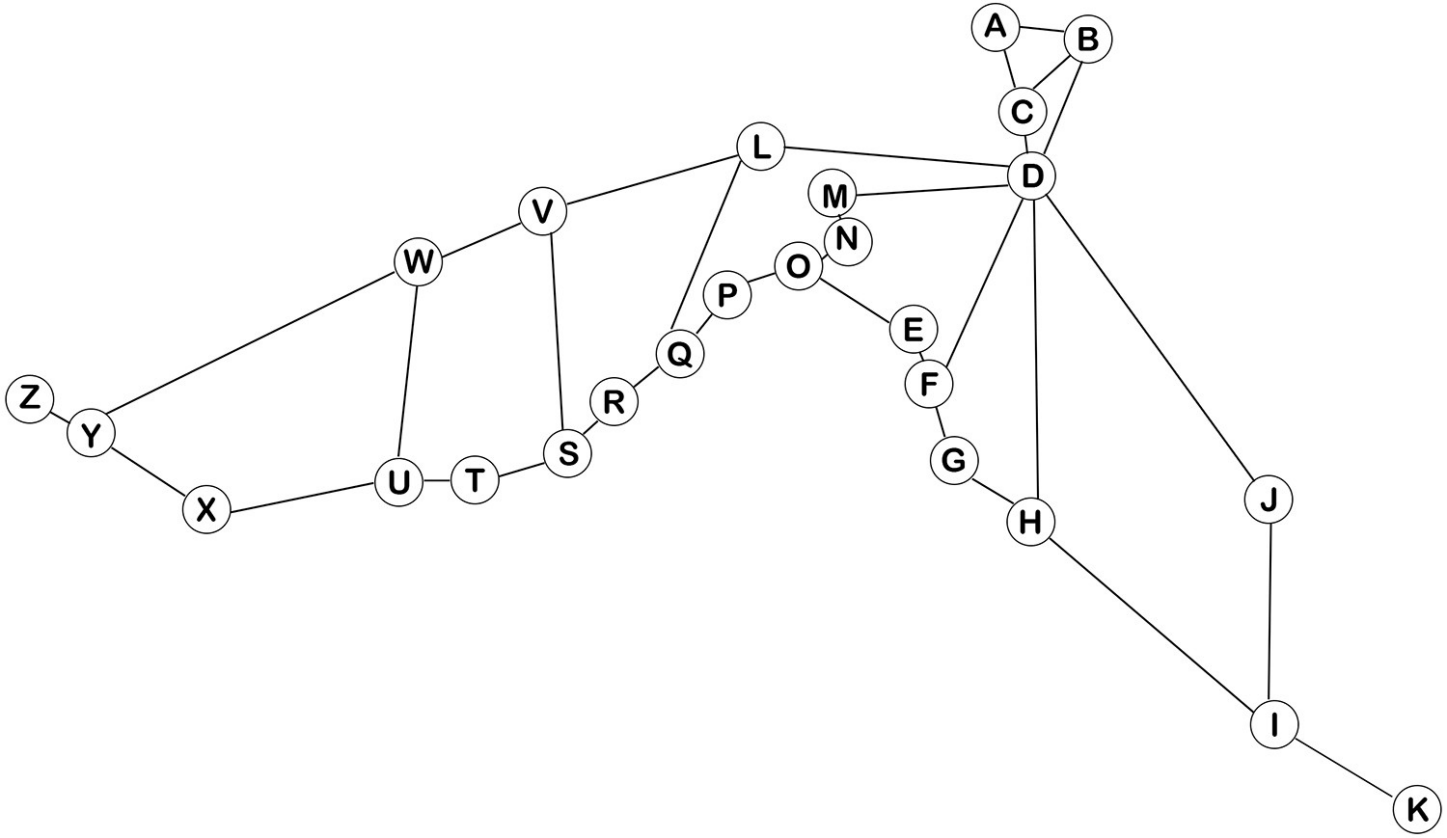
The schema and meeting points for ride-sharing.

Istanbul is chosen to be the simulation environment for the ride-sharing system. The city has a very wide public transportation web, yet it is one of the most crowded metropolitan cities in the world. There is also traffic congestion that keeps growing every day [39]. Therefore, the city may have a very large potential for ride-sharing. There are two main highways in Istanbul. A total of 26 meeting points are selected on these highways; the origins and destinations of all participants are assigned to these meeting points. In the simulation study, it is assumed that drivers will choose the shortest route for their convenience. Therefore, the shortest route between each meeting point is calculated using the real travel distances and Dijkstra’s Algorithm [40]. Travel times are calculated assuming that all vehicles travel at a constant speed. The schema and the defined meeting points are presented in Fig 6.

As mentioned, this paper aims to assess the effects of splitting drivers’ routes and setting JSS in the objective function. To do this, 4000 drivers and 4000 riders are randomly generated with an origin, a destination, either a capacity or rider count, social characteristics, and their weights. In the base case, 1000 riders with a rider count of 1 and 100 drivers with capacity of 3 are randomly selected from the generated data. The characteristics of the base case are summarized in Table 3.

**Table 3:** Characteristics of the base case.

Number of riders	1000
Number of drivers	100
Capacity of each driver	3 seats
Rider count of each rider	1 person
JSS Limit	-100
Number of allowable extra stops	10
Average travel distance	23.1 km
Average number of meeting points visited by drivers	6

To assess the sensitivity of the results to the parameters, different settings were created. In each setting, one of the parameters was changed. These parameters are the number of drivers, the number of riders, the lower bound of the JSS and the number of allowed extra stops. The results are evaluated using the following metrics: (a) the ratio of matched riders to all riders, (b) the ratio of matched drivers to all drivers, (c) the number of riders matched because of a route split, (d) average JSSs of the matched pairs, (e) the distance savings, as in system-wide distance savings as a fraction of the system-wide distances traveled when all participants travel alone.

### 5.2 Performance

Performance of an algorithm mostly depends on the sample size and the complexity of the algorithm. Since setting the objective function as maximizing JSS does not affect the sample size and complexity of the algorithm, only the effects of splitting drivers’ routes are considered for the computation time. The time complexity of the algorithm is given as O(mn), where m and n are the number of drivers and riders respectively. Therefore, the study also tested measuring the sensitivity of the computation time to different numbers of riders and drivers. The average and standard deviations of computation times in different settings are given in Table 4; each setting was tested 20 times. The matching algorithm was modeled in Python 2.7, and its performance was measured on a computer with an i5 2.7 GHz processor and 8 GB of RAM.

**Table 4:** Sensitivity of computation time to the route split and sample size.

	Case 1	Case 2	Case 3	Case 4	Case 5	Case 6
Splitting routes	Yes	No	Yes	No	Yes	No
Number of riders	1000	1000	1000	1000	100	100
Number of drivers	100	100	1000	1000	1000	1000
Average computation time (secs)	1.99	0.80	21.98	17.09	1.98	1.91
Standard deviation of computation time (secs)	0.10	0.04	0.31	0.18	0.02	0.03

Table 4 shows that the matching algorithm is feasible for use in real-life instances. Even with a large sample size (i.e., in Cases 3 and 4), the algorithm was solved in approximately 20 seconds. Splitting drivers’ routes brings computational burden to the algorithm; this burden results in an increase in computation times as expected, but using this feature allows the algorithm to be solvable in feasible times. The sample size selected for the simulation would be accumulated over several hours in real-life. The database can be updated with each incoming travel requests in practice. The computation time required for finding a successful match for a new single request can be less than 1 second.

### 5.3 Validation of heuristic solution

In this paper, a heuristic solution approach is proposed for solving the problem of ride-matching, because it is computationally prohibitive to solve the problem using IP. The proposed heuristic finds matches on a reasonably short notice, but the quality of solutions should be examined first before drawing any conclusions. To validate the proposed solution approach, the quality of solutions found by the heuristic solution is compared with the quality of solutions found by the IP that is presented in Section 3. Because it is unlikely for a large-scale problem to be solved using IP, the solution approaches are examined on small instances that consist of 50 riders and 50 drivers. The capacities of drivers are set to 1 for these instances, but multiple riders-single driver matches are possible because of the route split. The results are summarized in Table 5.

**Table 5:** Comparison of heuristic solutions and optimal solutions.

		Case 1	Case 2	Case 3	Case 4	Case 5	Case 6	Case 7	Case 8	Case 9	Case 10	Average
Heuristic	Sum of JSSs	2150	2490	2058	1811	1698	2035	1467	2222	1913	1919	1976.30
	Number of matched riders	21	24	22	20	20	22	19	23	19	23	21.30
	Number of matched drivers	21	21	20	18	17	20	15	22	19	19	19.20
Optimal	Sum of JSSs	2206	2962	2390	2251	2337	2696	2040	2565	2149	2661	2425.70
	Number of matched riders	21	27	24	20	23	24	19	25	21	24	22.80
	Number of matched drivers	21	27	23	20	20	24	19	23	21	23	22.10
Difference	Sum of JSSs	2.54%	15.93%	13.89%	19.55%	27.34%	24.52%	28.09%	13.37%	10.98%	27.88%	18.41%
	Number of matched riders	0.00%	11.11%	8.33%	0.00%	13.04%	8.33%	0.00%	8.00%	9.52%	4.17%	6.25%
	Number of matched drivers	0.00%	22.22%	13.04%	10.00%	15.00%	16.67%	21.05%	4.35%	9.52%	17.39%	12.92%

Table 5 shows that the average difference between heuristic solutions and IP solutions is less than 20%. The average difference in the number of matched riders is found to be 6.25%. Considering that the proposed heuristic solution approach can be used to solve more complicated and large-scale problems, the findings here suggest that this approach performs well for solving ride-matching problems.

### 5.4 Benefits of route split

To exploit drivers’ capacities, their routes are split after they are matched with a rider so that they can be matched again for the unmatched parts their routes. It is important to show how many riders are matched with drivers, whose routes were split before. Furthermore, it should be considered that these riders could be matched with other drivers if a route split was not allowed. To measure the benefits of splitting routes, the study analyses the distance savings and the total number of matched participants. A summary of the simulation results for different numbers of allowable extra stops are presented in Table 6.

**Table 6:** Simulation results for base case with different number of allowable extra stops.

	Number of allowable extra stops						
	0	1	2	3	4	5	6
Average number of riders matched before split of the route	258.75	247.2	244.3	245.2	237.3	241	235.2
Average number of riders matched after split of the route	0	39	74.7	94.9	102.7	104.9	110.1
Average number of all matched riders	258.75	286.2	319	340.1	340	345.9	345.3
Average number of all matched drivers	93.41	94.5	90.4	89.4	87.5	87.9	87.3
Ratio of matched riders to matched drivers	2.77	3.03	3.53	3.80	3.89	3.94	3.96

In Table 6, the column of zero allowable extra stops represents the algorithm without a route split feature. Extra stops are not recorded and not limited for this case. The results indicate that allowing route splitting causes a significant increase in the number of matched riders for the base case setting, where the capacity of each driver is set to 3. When one extra stop is allowed, the ratio of the matched riders to the matched drivers exceeds the capacities of the drivers. This shows that splitting drivers’ routes can cause a significant increase in the number of matches. Setting allowable extra stops greater than 3 does not significantly affect the number of matches for the simulation environment, where the average number of visited locations is 6. However, setting a higher number of allowable extra stops increases computational burden, even if it causes no significant increase in the number of matches. Therefore, the number of allowable stops should be selected carefully in order to maintain both a satisfactory number of matches and feasible computation times.

**Fig 7:**
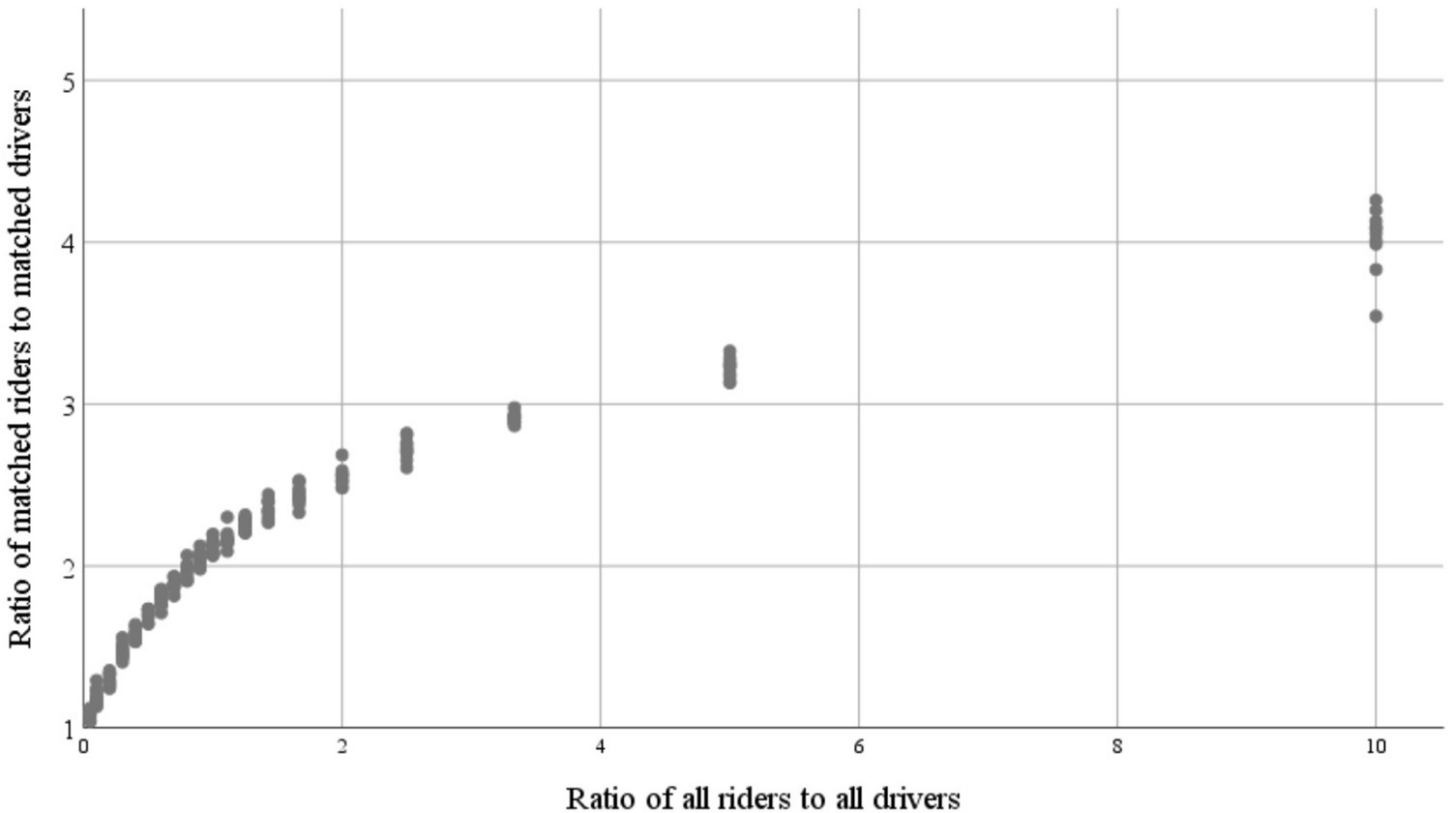
Ratio of matched riders to matched drivers versus all riders to all drivers.

It is suggested that route split would cause a significant increase in the number of matched riders when there is a shortage of drivers. The algorithm was also tested with different numbers of riders and drivers to measure the benefits of using a route split for various setups. The sensitivity of some results for a different number of riders and drivers is summarized in Table 7 and illustrated in Fig 7.

**Table 7:** Simulation results for different riders to drivers ratio.

	Ratio of number of riders to drivers						
	10:1	5:1	2:1	1:1	1:2	1:5	1:10
Number of riders	1000	1000	1000	1000	500	200	100
Number of drivers	100	200	500	1000	1000	1000	1000
Average number of riders matched before split of the route	238.48	410	709.5	871.85	468.4	193.4	96.6
Average number of riders matched after split of the route	110.6	105.4	78.8	56.35	12.3	1.2	0.1
Average number of all matched riders	349.08	515.4	788.3	928.2	480.7	194.6	96.7
Ratio of matched riders to all riders (%)	34.91	51.54	78.83	92.82	96.14	97.3	96.7
Average number of all matched drivers	88.02	160.1	309.2	437.7	282.9	150.1	81.3
Ratio of matched drivers to all drivers (%)	88.02	80.05	61.84	43.77	28.29	15.01	8.13
Ratio of matched riders to matched drivers	3.97	3.22	2.55	2.12	1.7	1.3	1.19

Table 7 shows that a route split makes a significant contribution to matching ratios, especially when the ratio of riders to drivers is relatively large. When the ratio of riders to drivers is greater than 5, the ratio of matched riders to matched drivers exceeds the capacities of drivers, which would not be possible without route splitting. Based on the simulation results shown in Fig 7, when the ratio of riders to drivers exceeds 3 for the base case setting, a route split causes the ratio of matched riders to matched drivers to exceed the capacities of drivers. When the ratio of riders to drivers is less than 1, splitting does not have a considerable contribution, because there are plenty of drivers for existing riders.

### 5.5 Effects of the joint socialness score

To create a successful ride-sharing system, a matching algorithm should consider not only system-wide benefits but also the concerns of potential participants who are social beings. As mentioned earlier in this paper, social compatibility of participants is scored using the JSS, and the objective function of the proposed matching algorithm is set to maximize the sum of JSSs. To assess the effects of utilizing the JSS in the objective function, the algorithm was also tested using a maximizing sum of distance savings as the objective function. To compare these two objectives, the base case setting was used, but the number of drivers was increased to 1000 in order to decrease the possible fluctuations due to heuristic behavior. The results are summarized in Table 8.

**Table 8:** Comparison of different objectives.

	Objective function	
	max JSS	max distance savings
Number of riders	1000	1000
Number of drivers	1000	1000
Number of matched riders	929.55	942.8
Total number of matched drivers	443.45	372.35
Average JSS	27.35	1.08
Standard deviation of JSS	0.59	0.65
Distance savings (%)	29.51	33.48
Standard deviation of distance savings (%)	0.67	0.69

Table 8 shows that compared to maximizing distance savings, the objective of maximizing the sum of JSSs results in a significantly larger average JSS value (i.e., 27.35 versus 1.08). Maximizing distance savings results in a slightly larger distance savings (i.e., 29.51% versus 33.48%). The matching algorithm assumes that a participant accepts the match offer from a participant with a low JSS if there is no better match offered to the participant. To maintain a certain level of satisfaction, each social parameters and/or JSS can be limited. JSS can be seen as the likelihood of a participant to accept the match found by a matching algorithm. Table 9 presents the sensitivity of the results to a JSS limit.

**Table 9:** Effects of JSS limit on the results.

	JSS Limit						
	-100	-20	0	20	40	60	80
Number of riders	1000	1000	1000	1000	1000	1000	1000
Number of drivers	100	100	100	100	100	100	100
Average number of matched riders	325.72	312.2	289.8	184.7	45.4	2.7	0.2
Average number of matched drivers	90.02	89.6	85.9	67.9	26.6	2.5	0.1
Average JSS	11.58	13.55	19.81	30.73	46.81	63.85	85

Table 9 shows that increasing the JSS limit causes an increase in average JSS of matches. The average JSS of all participants in the system is close to zero. Therefore, a dramatic increase is observed when the JSS limit exceeds zero. On the other hand, it should be noted that an increase in the JSS limit maintains a level of JSS by eliminating some matches, and thus an increase in the JSS limit causes a decrease in the number of matches.

## 6 Conclusions

This paper proposed a ride-matching algorithm that splits drivers’ routes for drivers to be matched again and includes social parameters of age, gender, employment and willingness to meet new people. The two-fold aim of the proposed algorithm is to serve more riders, especially in the case of a shortage of drivers, and to maximize user benefits in ride-matching so that more people would want to participate in a ride-sharing system. When a driver is matched for only part of his or her route, the proposed matching algorithm creates new travel requests using the unmatched part of the route. Thus, along the route, a driver can be matched with more riders. Furthermore, to increase the likelihood of a participant accepting the offered matches, a social compatibility score, JSS, is defined to measure the social compatibility of participants’ characteristics. In this way, a rider can be matched with the most compatible driver in the system.

The results of the simulation study indicate that the route split feature can substantially improve the performance metrics of a ride-matching algorithm (i.e., the number of matched riders and the distance savings). This feature would be most beneficial for achieving critical mass in ride-sharing when there are shortages of drivers. The results show that including route split feature in the matching algorithm causes 33% increase in number of matched riders. It should be mentioned that there is a trade-off between including this feature to the matching algorithm and the computation time. To address this issue, a heuristic was used to improve computation time performance. It was shown that the computation times are still feasible for real-life instances when 10 extra stops are allowed.

This study showed the benefit and cost in using the objective function of maximizing the sum of JSSs by comparing it with the objective of maximizing the sum of distance savings. Using a JSS caused a significant increase in average JSS, while it caused a slight decrease in the distance savings. The results suggest that with a small sacrifice from distance savings, the objective of maximizing the JSS provides more qualitative matches for participants.

In the future, it would be intriguing to investigate the performance of the proposed algorithm in different simulation environments. Improvements to the solution approach can also be discussed in order to obtain more qualitative results that are close to the optimal solution. In addition, this algorithm could be improved in various ways. For example, integrating public transportation systems to the ride-sharing system can be discussed to ensure that the proposed algorithm will be applicable in real-life instances.

## Supporting information

S1 Data (RAR)
